# Non-use of sunscreen among adults and the elderly in southern Brazil^☆^^☆☆^

**DOI:** 10.1016/j.abd.2018.10.002

**Published:** 2019-09-30

**Authors:** Elizabet Saes da Silva, Samuel Carvalho Dumith

**Affiliations:** Graduate Program in Health Sciences, Faculdade de Medicina, Universidade Federal do Rio Grande, Rio Grande, RS, Brazil

**Keywords:** Epidemiology, Skin neoplasms, Solar radiation, Sunscreening agents

## Abstract

**Background:**

One of the main prevention methods against skin cancer is the use of sunscreen; however, incidence of this disease has not declined despite prevention campaigns.

**Objective:**

Investigate the prevalence of non-use of sunscreen and its associated factors.

**Method:**

A population-based cross-sectional study with individuals aged 18 years or over living in the urban area. Conducted between April and July of 2016. Participants were interviewed about socioeconomic, demographic, and behavioral questions. Non-use of sunscreen was considered as the outcome. For multivariate analysis, Poisson regression with robust adjustment for variance was used.

**Results:**

Among the 1300 participants, prevalence of non-use of sunscreen was 38.2% (95% CI: 34.6–41.8). The variables independently associated with the outcome were male sex, older age, brown or black skin color, lower income, fewer years of education, no physical activity in leisure time, no medical consultations in the last year, and self-perception of health as regular or poor.

**Study limitations:**

The prevalence may be underestimated by reports of more use of sunscreen than actually used, which could increase the figure in the outcome.

**Conclusion:**

It was estimated that about four out of ten adults and elderly do not use sunscreen in this sample. Prevention strategies are needed to advance health policy and ensure that sun protection options are easily accessible.

## Introduction

Excessive exposure to ultraviolet (UV) radiation can generates oxidative stress in skin cells, resulting in direct damage to nucleic acids and proteins, leading to genetic mutation or cells death (apoptosis)^1^, ^2^, ^3^. In view of this, this behavior is an established risk factor for skin cancer^1^, ^2^. Therefore, formulated products were developed to protect the skin from UV radiation, known as sunscreens^4^, ^5^.

There are two types of sunscreens, organic and inorganic. The former absorb UV radiation, while the latter disperse and reflect UV radiation, blocking UVA and UVB rays^5^, ^6^, ^7^, ^8^. Zinc oxide (ZnO) and titanium dioxide (TiO_2_) are physical sunscreens, which have ten times larger particles (size range of 200–500 nm), and, for this reason they achieve better light reflection and dispersion^6^, ^7^, ^8^. However, it should be noted that these components have been investigated for inducing the formation of peroxides, free radicals, and other reactive oxygen species (ROS) when irradiated with UV light, promoting DNA damage^9^, ^10^, ^11^. Notwithstanding, sunscreen is still a primary prevention method against skin cancer, supported by evidence from a clinical trial conducted by Green et al., in which the regular use of sunscreen reduced the incidence of melanoma^12^.

Several epidemiological studies investigated sunscreen use to prevent skin cancer^13^, ^14^, ^15^, ^16^, ^17^, ^18^, ^19^. The prevalence of non-use of sunscreen varies from 9.9% up to 68%^13^, ^14^, ^15^, ^16^, ^17^, ^18^, ^19^. In Brazil, a study conducted in the city of Pelotas (RS) observed prevalence of non-use of sunscreen of 39.2%, 69.8%, and 86.3% at the beach, during sports, and at work, respectively^20^. In a research in the municipality of Carlos Barbosa (RS), a prevalence of 25.7% for this behavior was found^21^. Another study conducted in 15 capitals of Brazil showed frequencies around 92% among men and 76% among women^22^.

Despite many awareness campaigns to protect against skin cancer, incidence of this disease has not reduced^23^. In Brazil, few population-based studies have investigated the prevalence of sunscreen use^20^, ^21^, ^22^ .Thus, this study aimed to investigate the prevalence of non-use of sunscreen and its associated factors among the adult and elderly population of a municipality in southern Brazil.

## Methods

This was a population-based study with a cross-sectional design. It was conducted in 2016 in the urban area of Rio Grande, a coastal city in southern Brazil. This municipality has approximately 200,000 inhabitants (96% urban), with a Human Development Index (HDI) of 0.744. Seasons are well-defined and the mean temperature ranges from 12.9 to 23.6 °C^24^, ^25^. UV radiation varies throughout year. In winter months, the UV index is around 4 (moderate), while in warmer months it reaches 13 (extreme)^26^. This study was part of broader research project entitled “Health of the Rio Grande Population,” which aimed to assess different health aspects of the population of Rio Grande, Rio Grande do Sul, Brazil.

A descriptive sample size calculation was conducted considering following parameters: 95% confidence level, 10% expected prevalence, and margin of error of 2%. This study also accounted for design effect (deff) and losses/refusals, adding 50% and 10% respectively, resulting in 1420 individuals. Sample size calculation for associated factors was performed considering following parameters: 80% power, 95% confidence level, 10% expected prevalence, and prevalence ratio of 2.0. The authors added 15% to the sample size to estimate the multiple correlations in the adjusted analysis, where the independent variable, the dependent variable, and some intervening variables are the potential confounders, and 10% for losses/refusals. Thus, this study needed to include at least 1423 individuals.

A multiple-stage sampling strategy was used to obtain a representative sample of the urban population of the city, based on data of the 2010 Population Census^24^. Firstly census tracts were sampled, secondly households, and lastly individuals.

For the first stage, to compose a sample of 1423 individuals, it would be necessary to sample 710 households (average of two individuals per household), corresponding to 72 census tracts (considering that the authors expected to survey at least ten households per census tract)^24^. Therefore, a list was created with all the households of Rio Grande (77,835). This list was organized in descending order according to the monthly income of the head of each family. Subsequently, the first household was randomly selected as a starting point. After this step, one in every 1080 households was systematically selected (77,835/72). At the end of this process, a list of 72 households was obtained, of which only the information about the census tracts in which they were located was saved, resulting in a list of 72 census tracts.

In the second stage, households from the list of 72 census tracts that was previously created were randomly selected. Similarly to the first stage, these 72 census tracts were transformed into a list of 23,439 households that belonged to these tracts. The authors started by randomly selecting a first household as a starting point. Afterwards, one in every 32 households was systematically selected (23,439/710). This process resulted in 711 households sampled. Finally, all individuals of the selected households that fulfilled the eligibility criteria composed the sample of this study.

Data were collected between April and July of 2016. Interviewers used a pre-coded questionnaire with closed questions. Fieldwork supervisors conducted telephone interviews with 10% of the sample, in order to control the quality of information and to overcome possible fraud in application of the instrument. The kappa coefficient was 0.80, considered sufficient. Data were revised, coded, and then double-entered using EpiData software, v. 3.1.

Inclusion criteria were being 18 years of age or older and living in the urban area. Exclusion criteria were institutionalization in nursing homes, hospitals, or prisons, or having a physical and/or mental disability that prevented answering the questionnaire.

The outcome was assessed through the question “Do you usually use sunscreen?” Respondents had three choices, namely: “no,” “yes, only in the summer,” and “yes, all year round.” Participants who answered negatively were considered the numerator to calculate the prevalence of the outcome.

The independent variables were as follows: sex (male/female), age (18–39/40–59/≥60), skin color (white/brown/black), marital status (single/married/divorced/widow[er]), time living in the neighborhood (<5 years/5–10 years/>10 years), education (0–8 years/9–11 years/≥12 years), wealth index in terciles (from lowest to highest), smoking (non-smoker/smoker), physical activity during leisure (no/yes), body mass index (normal/excess weight), medical consultation in the last year (no/yes), self-perception of health (excellent/very good/good/regular/poor), and history of skin cancer (no/yes).

The variable wealth index was used as an indicator of socioeconomic status^27^. This variable took into account household characteristics (source of drinking water, number of sleeping rooms, and toilets) and assets (car, computer, internet, landline telephone, microwave, washing machine, clothes dryer, and DVD player). From these 11 items, a principal component analysis was performed, where the first component was extracted, which explained 30.1% of the variability of all items (eigenvalue = 3.31).

The exposure variable “history of skin cancer” was assessed through following question: “Has any doctor ever told you that you have cancer?” When affirmative, respondents were also questioned “Where (or what type of cancer)?” Those who responded positively to some type of skin cancer were considered exposed. Body mass index (BMI) was obtained by calculating the self-reported weight and height, considering BMI 18.5–24.9 kg/m^2^ as normal and BMI 25–30.0 kg/m^2^ as excess weight.

The variable “time in the neighborhood” was used in the model as a proxy of how long the subjects had resided in the city, since it is a port city with intense migration.

Statistical procedures were performed using the software STATA v. 11.2 (StataCorp LP – College Station, Texas, United States). Univariate analysis was conducted to describe the sample regarding all variables of interest. Bivariate analysis was used to calculate prevalence of the outcome according to independent variables. Multivariate analysis was performed according to a hierarchical model or conceptual model of analysis to determine the order of entry of the variables in the analysis^28^. It was composed of four levels: the first level comprised social variables (sex, age, skin color, and marital status); the second contained economic and demographic variables (time in the neighborhood, education, and wealth index); the third was composed of behavioral variables (smoking, physical activity during leisure, overweight), and the fourth consisted of health variables (medical consultation, self-perception of health, and history of skin cancer). Variables with a *p*-value ≤ 0.20 were maintained in the model as a strategy to control for confounding. Poisson regression with robust adjustment for variance was used to calculate crude and adjusted prevalence ratio (PR) and its corresponding 95% confidence interval (95% CI), and the *p*-values. Wald's test was used for heterogeneity (dichotomous or nominal exposures) and for linear trend (ordinal exposures). The confidence level was set at 95% and the significance level, at 5%.

This research was approved by the Research Ethics Committee in the Health Area of the Federal University of Rio Grande (registration No. 20/2016). All ethical principles established by the National Health Council in Resolution 466/12 were respected. Participants were informed of their right to refuse participation and about confidentiality procedures. Those who agreed to participate in the study signed the informed consent.

## Results

Of the 1423 eligible individuals, 1300 participated in the study, a response rate of 91.0% and 9.0% losses (6.9% refusal and 2.1% not found). Table 1 presents the sample description. The proportion of female individuals was slightly higher (56.6%), while white skin color was predominant (83%). Almost half (42%) had eight years or less of education. 82% were non-smokers, 66.6% reported not practicing physical activity during leisure, and 61.6% had excess weight. Self-perception of health was described as good by 44.7% and as being regular or poor by 33.9%. The majority of participants (79.9%) had had a medical consultation in the last year. Prevalence of non-use of sunscreen was 38.2% (95% CI: 34.6%–41.8%), and the deff and intraclass correlation coefficient were 1.79 and 0.038, respectively. Some variables such as schooling, skin color, index of goods, physical activity during leisure, and medical consultation during the year presented missing data.

**Table 1:** Distribution of the sample of adults and elderly of the municipality Rio Grande, RS, Brazil, 2016 (*n* = 1300).

Variable	*n*	%
*Sex*		
Male	564	43.4
Female	736	56.6

*Age (years)*		
18–39	508	39.1
40–59	477	36.7
≥60	315	24.2

*Skincolor*^a^		
White	1077	83.0
Brown	103	7.9
Black	108	8.3

*Marital status*		
Single	602	46.3
Married, divorced, or widow(er)	698	53.7

*Time in the neighborhood (years)*		
<5	335	25.8
5–10	500	38.4
>10	465	35.8

*Education (years)*		
0–8	543	41.8
9–11	400	30.8
≥12	355	27.4

*Wealth index (tercile)*		
1st (lowest)	447	34.4
2nd	419	32.3
3rd (highest)	433	33.3

*Smoking*		
No	1066	82.0
Yes	234	18.0

*Physical activity during leisure*		
No	862	66.6
Yes	433	33.4

*Body mass index (BMI)*^b^		
Normal	477	38.4
Excess weight	765	61.6

*Medical consultation in the last year*		
No	260	20.1
Yes	1037	79.9

*Self-perception of health status*		
Excellent/very good	278	21.4
Good	582	44.7
Regular/poor	440	33.9

*History of skin cancer*		
No	1292	99.4
Yes	8	0.6

*Non-use of sunscreen*		
No	496	38.2
Yes	804	61.8
Total	1300	100.0

a Ten individuals (0.8%) were excluded from this analysis (yellow skin color or indigenous).

b 58 individuals did not report information for BMI.

Table 2 shows the prevalence of the outcome according to independent variables and results of multivariate analysis. Men had a higher probability of non-use of sunscreen compared to women (PR = 1.82; 95% CI: 1.59–2.07). Age, skin color, and self-perception of health were positively associated with the outcome, so that participants over 40 years of age (PR = 1.26, 95% CI: 1.07–1.98), with brown (PR = 1.34, 95% CI: 1.12–1.61) or black skin color (PR = 1.57, 95% CI: 1.32–1.88), or with self-perception of health as regular or poor (PR = 1.60, 95% CI: 1.24–2.06) showed higher probability of non-use of sunscreen.

**Table 2:** Analysis of non-use of sunscreen in adults and elderly in the city Rio Grande, RS, Brazil, 2016 (*n* = 1300).

Variable	% non-use of sunscreen	Crude		Adjusted	
		PR (95% CI)	*p*-Value	PR (95% CI)	*p*-Value
*Sex*			<0.001		<0.001
Male	50.9	1.79 (1.57–2.04)		1.82 (1.59–2.07)	
Female	28.4	1.00		1.00	

*Age (years)*			<0.001^a^		<0.001^a^
18–39	31.3	1.00		1.00	
40–59	35.9	1.15 (0.98–1.34)		1.26 (1.07–1.98)	
≥60	52.7	1.68 (1.42–1.99)		1.96 (1.67–2.31)	

*Skin color*			<0.001		<0.001
White	35.5	1.00		1.00	
Brown	49.5	1.40 (1.14–1.71)		1.34 (1.12–1.61)	
Black	53.7	1.51 (1.26–1.81)		1.57 (1.32–1.88)	

*Marital status*			0.210		0.105
Single	36.2	1.00		1.00	
Married, divorced, or widow(er)	39.8	1.10 (0.95–1.28)		1.14 (0.97–1.33)	

*Time in the neighborhood (years)*			<0.001		0.327
<5	34.6	1.00		1.00	
5–10	33.4	0.97 (0.77–1.21)		0.96 (0.77–1.20)	
>10	45.8	1.32 (1.11–1.58)		1.07 (0.90–1.28)	

*Education (years)*			<0.001^a^		<0.001^a^
0–8	55.8	3.14 (2.35–4.21)		2.51 (1.86–3.39)	
9–11	32.0	1.80 (1.32–2.46)		1.61 (1.18–2.21)	
≥12	17.8	1.00		1.00	

*Wealth index (tercile)*			<0.001^a^		0.006
1st (lowest)	51.2	1.79 (1.48–2.16)		1.27 (1.06–1.52)	
2nd	34.1	1.19 (0.95–1.50)		1.03 (0.84–1.27)	
3rd (highest)	28.6	1.00		1.00	

*Smoking*			0.001		0.289
No	36.4	1.00		1.00	
Yes	46.2	1.27 (1.10–1.46)		1.09 (0.93–1.27)	

*Physical activity in leisure*			<0.001		0.002
No	43.0	1.52 (1.26–1.83)		1.31 (1.10–1.55)	
Yes	28.4	1.00		1.00	

*Body mass index (BMI)*			0.183		0.347
Normal	35.4	1.00		1.00	
Excess weight	39.2	1.11 (0.95–1.29)		1.07 (0.93–1.22)	

*Medical consultation in the last year*			<0.001		0.008
No	49.6	1.40 (1.21–1.63)		1.25 (1.06–1.48)	
Yes	35.4	1.00		1.00	

*Self-perception of health status*			<0.001^a^		<0.001^a^
Excellent/very good	23.0	1.00		1.00	
Good	36.9	1.61 (1.19–2.17)		1.23 (0.93–1.62)	
Regular/poor	49.3	2.14 (1.64–2.79)		1.60 (1.24–2.06)	

*History of skin cancer*			0.246		0.319
No	38.3	1.00		1.00	
Yes	12.5	3.07 (0.46–20.7)		2.72 (0.37–19.8)	

PR, prevalence ratio; 95% CI, 95% confidence interval.

a Wald's test for linear trend.

Respondents who had not had a consultation with a physician in the last year (PR = 1.25, 95% CI: 1.06–1.48) and who did not practice physical activity during leisure (PR = 1.31, 95% CI: 1.10–1.55) also had a higher probability of non-use of sunscreen. Nonetheless, it was identified that the lower the educational level (*p* < 0.001) and the lower the wealth index (*p* = 0.006), the higher the probability of non-use of sunscreen. The variables time in the neighborhood, smoking, marital status, BMI, and history of skin cancer were not associated with the outcome.

## Discussion

This is the first population-based adult sample study in the municipality of Rio Grande that aimed to assess the prevalence of non-use of sunscreen and its associated factors. A frequency of non-use of sunscreen (38.2%) was found, also showing that men had higher probability than women of having this behavior. Scientific literature points out that men are less concerned with the harmful effects of UV radiation and that women use sunscreen more frequently, probably due to the a higher sense of care for their health and esthetics^13^, ^15^, ^16^, ^17^, ^22^.

The present analysis showed that the greater the age, the higher the probability of non-use of sunscreen (*p* < 0.001). A plausible explanation would be that the older individuals might view exposure to UV radiation as a health benefit, thus not having the habit of using sunscreens, since in Brazil this behavior began to be introduced in the 1980s, when the first sunscreens appeared^29^, ^30^. Nonetheless, these individuals may have sought other means of protection such as hats, clothing and shade, as they did not have a history of skin cancer in this sample^16^, ^18^.

Brown and black skin colors were positively associated with the outcome. The darker the skin color, the higher the probability of non-use of sunscreens. It is well known that the higher the level of melanin in the skin, the higher the natural protection against UV radiation^31^. Despite this natural protection, there is evidence that darker skins can develop a type of skin cancer, squamous cell carcinoma^32^. Skin cancer is less prevalent in people with darker skin color than in the white population, but usually presents at a more advanced stage, since this group does not have adequate protective behavior, as observed in this population, which may increase the morbimortality associated with this pathology^33^.

Physical activity during leisure is mainly practiced outdoors, increasing exposure to UV radiation, which requires specific care^22^. Prevalence of non-use of sunscreen among those physically active in leisure was 28.4%, which was lower than among those not considered physically active in this domain (43%). A study conducted in the municipality of Pelotas showed that about 60% of individuals did not use sunscreen during the practice of sports, which two-fold greater when compared to present results^20^. Thus, the practice of these activities should be the moment to establish a strategy of primary prevention^22^.

The population under study had a high proportion of physically inactive (66.6%) and overweight (61.6%) individuals. Prevalence of non-use of sunscreen among those with excess weight was 39.2%, similar to those with normal BMI (35.4%, *p* = 0.347). Furthermore, Szklo et al., in their household survey conducted in 15 Brazilian capitals, showed that in Porto Alegre the prevalence of non-use of sunscreen was 21% among those with BMI < 25 kg/m^2^ compared to 25.9% in those with BMI ≥ 25 kg/m^2^^22^. The prevalence of non-use of sunscreen in the city of Porto Alegre as well as in the city of Rio Grande did not show differences between people with normal BMI and those with excess weight, although the city of Rio Grande presented higher frequencies.

This study identified a low proportion of smokers (18%), a subgroup that had a prevalence of non-use of sunscreen of 46.2%, although without statistical significance when comparing to their non-smoker counterparts (*p* = 0.289). Another characteristic of this population was the high frequency of medical consultations, about 80%. Prevalence of non-use of sunscreen in this subgroup was 35.4%, lower than those who had not had a medical consultation (49.6%), a difference that was statistically significant (*p* = 0.008). It is possible that in those medical consultations individuals received information and guidance regarding the use of sunscreen^34^. Nonetheless, those who had medical consultation and reported non-use of sunscreen might not have had the same instructions^34^.

History of skin cancer was investigated in this study by self-report of having had skin cancer diagnosed by a physician. Only eight (0.6%) individuals in the sample reported having had skin cancer. Due to this low frequency, there was a lack of statistical power (11%) to analyze this variable.

Non-use of sunscreen was inversely associated with education, as well as with socioeconomic status. The high prevalence of non-use of sunscreen among those with fewer years of education (55.8%; *p* < 0.001) might be explained to the lack of knowledge about the harmful effects of exposure to UV radiation and protection methods. In addition, it is possible that the high frequency of non-use of sunscreen among the poorest subgroup (51.2%; *p* = 0.006) is due to financial restrictions that prevents them from buying and using sunscreens^14^. These results are in line with previous studies^13^, ^14^, ^15^, ^16^, ^20^. Therefore, the Brazilian government is considering a bill that obligates the provision of sunscreen in the public setting to increase access to it, especially among the poorest^20^. Moreover, these individuals may be taught to use other prevention and protection methods, such as seeking shade, and using hats, parasols, and appropriate clothes^15^, ^16^.

Latitude must also be considered. The UV radiation contains much more UVA than UVB energy, particularly at high latitudes^35^. While UVB radiation decreases with distance from the Equator, UVA radiation has its higher concentration between the 20° and 40° parallels^22^, ^36^. The municipality of Rio Grande is situated at 32.2° latitude, where the highest concentrations of UVA radiation can be found^37^. Thus, individuals of this city who reported non-use of sunscreen may be at greater exposure to these high levels of radiation.

This study has some limitations. The first is regarding self-reported measures. It is possible that the prevalence was underestimated because the participants might have reported higher use of sunscreens than they actually did. Secondly, data about other protection methods and levels of exposure to UV radiation were not collected. Thirdly, the cross-sectional design prevents the establishment of causal relationships. Lastly, extrapolation of the results to the general population should be conducted with caution. The data were collected in autumn and winter, with less UV radiation than would be expected in the summer. Furthermore, the seasons and climate of Rio Grande may differ from other municipalities in Brazil and in the world.

## Conclusions

Based on the results of this study, it is estimated that four out of ten adults and elderly of the municipality of Rio Grande do not use sunscreens. The risk groups were: men, the elderly, those with brown or black skin, those with less education, those without physical activity during leisure, those who had not attended a medical consultation in the last year, and those who had perceived their health as regular or poor. Prevention strategies are needed to advance health policy and ensure that sun protection options are easily accessible. Educational campaigns regarding the benefits and harms related to intensity and time exposed to UV radiation should also be promoted.

## Author's contribution

Elizabet Saes da Silva: Elaboration and writing of the manuscript; critical review of the literature.

Samuel Carvalho Dumith: Statistical analysis; approval of the final version of the manuscript; conception and planning of the study; obtaining, analyzing and interpreting the data; effective participation in research orientation; critical review of the manuscript.

## Financial support

The study received funding from FAPERGS (Foundation for Research Support of the State of Rio Grande do Sul, Brazil), grant ARD/PPP/2014, case 16/2551-0000359-9.

## Conflicts of interest

None declared.
